# User-centered visual explorer of in-process comparison in spatiotemporal space

**DOI:** 10.1007/s12650-022-00882-3

**Published:** 2022-11-09

**Authors:** Dong Yu, Oppermann Ian, Liang Jie, Yuan Xiaoru, Nguyen Quang Vinh

**Affiliations:** 1 School of Computer Science, University of Technology Sydney, Ultimo, Australia; 2 Key Laboratory of Machine Perception (Ministry of Education), and School of AI, Peking University, Beijing, China; 3 National Engineering Laboratory for Big Data Analysis and Application, Peking University, Beijing, China; 4 School of Computing, Engineering and Mathematics, Western Sydney University, Sydney, Australia

**Keywords:** Comparative visualization, User-centered, Progressive exploration, Spatiotemporal features, COVID-19

## Abstract

**Abstract:**

We propose a user-centered visual explorer (UcVE) for progressive comparing multiple visualization units in spatiotemporal space. We create unique unit visualization with the customizable aggregated view based on the visual metaphor of flower bursts. Each visualization unit is encoded with the abstraction of spatiotemporal properties. To reduce user cognition load, UcVE allows users to visualize, save, and track in-the-process exploration results. In coordination of storage sequence and block tracking views, UcVE can facilitate comparison with multiple visualization units concurrently, selected from historical and current exploration results. UcVE offers a flexible geo-based layout, with aggregation functions and temporal views of the timeline with categorized events, to maximize the user’s exploration capabilities. Finally, we demonstrate the usefulness by using COVID-19 datasets, case studies with different user scenarios, and expert feedback.

**Graphical abstract:**

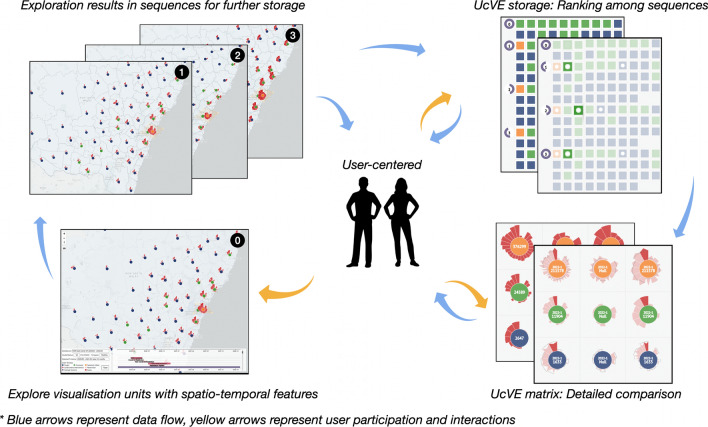

## Introduction

In the era of big data, the explosive growth of multivariable data, particularly with both spatial and temporal attributes, poses many challenges to data analysis tasks. In particular, a growing number of visual analysis systems are required not only to provide flexible exploration but also explicitly to provide comparisons for multiple data elements during the exploration process. Taking the current global pandemic as an example, federal, state, and local community public health officials synthesize highly disparate data to facilitate timely communication with the public and inform decisions regarding policies to protect public health. Since the early outbreak, visualization has played a central role in dealing with public health risks. An astonishing number of visual representations have been prepared to reflect the phenomenon and its effects worldwide. However, as the amount and complexity of infectious disease data increases, the comparative analysis of epidemiology data according to various perspectives, e.g., spatial and temporal information, still remains a fundamental challenge.

Based on challenges and research gaps of spatiotemporal data mining (STDM) summarized by Ali et al. in 2021 (Hamdi et al. 2022), we noticed 5 factors needed to be addressed in exploring spatiotemporal space:

1. Spatiotemporal object relationships that are complex and implicit.
2. STDM requires interdisciplinary effort and integration of various heterogeneous datasets and multiple data mining algorithms.
3. Spatiotemporal region discretization problem caused by the scale and the zoning effects on the data mining results.
4. Data characteristics such as heterogeneity and dynamicity.
5. Further efforts needed in STDM for data representations, advanced modeling, visualization, and comprehensiveness.

To allocate these challenges, we cooperated with Lan and further developed an original User-centered Visual Explorer (UcVE) based on unit visualization with scalable aggregated views. The spatiotemporal features are encoded in each visualization unit for their variation. UcVE allows users to view, save, and track the outcomes of in-progress exploration to lessen the cognitive load on the user. UcVE can facilitate concurrent comparison with multiple visualization units, which are selected from historical and current exploration results in coordination with storage sequence and block tracking views. To maximize the user’s exploration ability, UcVE provides a flexible geo-based layout, aggregation functions, and temporal views of the timeline with categorized events. Using COVID-19 datasets, we present case studies in various user contexts. We also worked with domain experts to discuss our case findings and provide expert feedback for evaluations.

Our paper makes the following main contributions in three orientations:

- *Design-oriented: A visual metaphor of unit visualization with the customizable aggregated view* to expand the visual representation scalability (C1, C5). The UcVE maps unit visualization with the encoded abstraction of spatiotemporal information in three statuses: single, auto-clustered, and custom-merged (C2), which allows for easy tracking and details on dynamic demand for each individual variation (C4), making exploration easier.
- *Human-oriented: User-centered Progressive exploration approach with saving and tracking in-process visualization results* to ease user cognition workload. Users can record each interacted result on a map as a storage sequence for further iterative callback and exploration to deal with spatiotemporal region discretizations (C3, C4); ranking and target tracking can also be done interactively between different storage sequences (C5).
- *Comparison-oriented: Comparative Visualization with user-selected multiple visualization units concurrently,* breaking to geo-barriers to explore implicit relationships among units (C1, C3). Combined with other visualization views and interactions, a comparison matrix view enables a detailed comparison of spatiotemporal attribute values among multiple visualization units in a scalable manner (C5).

The remainder of the paper is organized as follows: The related work is listed in Sect. 2. We list our design requirements in Sect. 3. In Sect. 4, we elaborate on the visual design for UcVE based on the extracted requirements. Section 5 describes COVID-19 as an example of spatiotemporal dataset. Section 6 is structured for COVID-19 datasets to demonstrate the workflow of the UcVE-based visual analytics system. We demonstrate the capability of our approach through three case studies in Sect. 7 and a discussion with domain experts in Sect. 8. We discuss the limitations and future work in Sect. 9. Finally, we summarize the conclusion in Sect. 10.

## Related work

This section presents relevant studies on visual exploration of spatiotemporal data and visual analytics of COVID-19 data.

### Visual exploration of spatiotemporal data

A summary of geospatial content by Yoshizumi et al. (2020) indicates that there were 94 of 220 papers in recent IEEE VIS publications used geospatial data. A variety of VA methods and tools have been developed to visually make sense of geospatial data (Andrienko et al. 2016). Among them are the analysis of the origin-destination (Zhou et al. 2018; Liu et al. 2020), the vehicle trajectory (Scheepens et al. 2011; Al-Dohuki et al. 2016; Liu et al. 2020), the vehicle flow analysis (Weng et al. 2020), and etc (Nakaya and Yano 2010; Kerry et al. 2010; Hagenauer et al. 2011; Levine et al. 2013; de Melo et al. 2018).

The spatiotemporal features of geospatial datasets complicate matters of analysis (White et al. 2010). In contrast to simple geographic information path or sampling point analysis (Ratcliffe 2000, 2006), time-varying attributes in especially large-scale dataset (Deng et al. 2021) bring a high level of uncertainty in the dissemination and change of geographic information, and different data attributes introduce a large number of cascading relationships, making it difficult to analyze patterns underlying datasets using traditional visual methods. Time curves (Bach et al. 2015) provide a visual method to illustrate and reveal informative patterns in a range of different spatiotemporal datasets. Brehmer et al. (2016) surveyed several timelines and designed a hybrid timeline representation that combines different timeline representations in a three-dimensional space.

As a result, in addition to a series of spatiotemporal visualization encodes on the map (Wang et al. 2020), the researchers also purposed the KDE (Silverman 2018) (kernel density estimation), which can help with the problem of uneven distribution density. Hurter et al. (2012) provided a KDE-based visual clustering approach to depict clutters in complex graph drawing. Since then, various clustering method have been applied to spatiotemporal data to depict clustering by different parameters, which can effectively reduce visual overlapping issues (Maciejewski et al. 2009; Lukasczyk et al. 2015; Aljrees et al. 2016). DDLVis (Li et al. 2021) provides a VA system with applied peak-based kernel density estimation method to produce the data distribution for the spatiotemporal data. Compass (Deng et al. 2021) is presented for analyzing the dynamic causality in urban time series. In addition to clustering algorithms and other AI algorithms (Andrienko et al. 2010; Zhou et al. 2019; Jamonnak et al. 2021) to assist analysis, spatiotemporal data analysis also necessitates human–computer interaction, which are given as follows: Andrienko et al. combined multiple map screenshots with SOM method for analyzing multiple map views, and Lee et al. present a VA system (Lee et al. 2019) that used short-term memory model to forecasting, as well as support users to inspect each traffic congestion caused in multiple views. A bus network-based VA system (Weng et al. 2020) supports to visually compare each route parameters in spatiotemporal aspects.

Most visual analysis systems based on spatiotemporal data today can help people reduce overlapping and dimensionality using AI algorithms (Brunsdon et al. 2007; Hagenauer et al. 2011; Li et al. 2017; de Melo et al. 2018), as well as record and compare the results of multiple analyses by taking screenshots or displaying parameters side by side. However, few of them can support the flexible expansion of the interaction between each storage, like tracking and comparing spatiotemporal features among different storages, which is reducing the capabilities of each storage significantly. As a consequence, the support system should not only include algorithms for parsing spatiotemporal data, but also some exploration strategies for storing as much information as possible from each user interaction for later recall and comparison.

### Visual analytics of COVID-19 data

With the pandemic as a backdrop, there have already remained two main kinds of COVID-19 data research in visualization: micro-perspective in scientific visualization with this virus that is analyzed from a biomedical perspective and combined with clinical medicine (Nguyen et al. 2021; Li et al. 2020; Liu et al. 2021). And macro-perspective in multiple related COVID-19 datasets, which contain such infection cases, recovery, and mortality rates with COVID and connected with social factors like geographical (Goetschel et al. 2021), social media and journalism (Yu et al. 2021; Leite et al. 2020), trajectory of human mobility (Yang et al. 2022), and other factors (Antweiler et al. 2021; Gharizadeh et al. 2020; Hua et al. 2020).

Since the outbreak of the epidemic, sorts of visualization systems such as JHU CSSE (Dong et al. 2020) and WHO (WHO 2021) have been gradually developed, and various countries have also developed numerous applications according to their own pandemic situations. Kamel Boulos and Geraghty (2020) summarized a series of practical online/mobile GIS and mapping dashboards and applications in 2020, and later in 2021, Zhang et al. (2021) summarized 668 visualizations related to COVID-19 and proposed a framework for analyzing the visualization of the epidemic crisis, to clarify the problems that need to be solved in each framework level. Among them, there are questions about what kind of COVID data to use, to whom, and how to present the data. This also directly defines the research basis for us. Jiang et al. (2020) presented a knowledge graph system to interactively explore epidemic situations. Yang et al. noticed that based on crowd movement and control measures may have an impact on the epidemic, so they proposed EpiMob (Yang et al. 2022), a VA system which simulates the changes in human mobility and infection status. However, most of these visualization methods focus on displaying data to specific populations (Jadhav et al. 2021; Wu et al. 2021) or using infectious disease models (Afzal et al. 2020) to analyze and predict data (Srabanti et al. 2021), whereas GIS-based geographic information systems can only pre-mark sampling points on the map and cannot interact, limiting the human–computer interaction of extended analysis capabilities and making joint analysis difficult for decision makers.

## Requirements analysis

Our goal is to create a visual approach that allows users to progressively explore and compare multiple visualization units with different spatiotemporal properties.

We know from previous research that analyzing spatiotemporal data is one of the most difficult aspects of visual analysis, so how to clearly and completely abstract spatiotemporal features is our main design requirement. Other related properties, for example, multivariable and textual data, are usually present with spatiotemporal data. In addition to visualizing all spatiotemporal features, we must consider designing and implementing user-based interaction methods that are applied to every individual user scenario to assist users in in-depth further complex spatiotemporal dataset analysis.

This work aims to focus on the majority of analytical tasks in spatiotemporal data which can be abstracted their spatial location accompanied with numerical attribute values in their temporal features. In Sect. 5, we introduced the COVID-19 crisis context as an example of typical spatiotemporal datasets and applied them to UcVE. We also discussed the design requirements in the collaboration with domain experts analysts in the NSW government and distilled the requirements as below.

- *R1: Draw encoded visualization units in a reasonable manner* The fundamental method of mapping the geographic coordinates of units to a map makes a geo-based layout easy to implement but to encode the adjustable temporal features of each unit while displaying the geographic coordinates remains a design challenge that needs to be addressed. As a current instance, the analysts in NSW Government released a visual interface (Government 2022) for COVID-19 basic statistics for each geo-spot which used colored heat-polygons on a playable timeline rather than abstracted as visualization units for each spot. This consequence makes inspection worse when exploring polygons with large size differences on different scales, and the detailed values are not evident to map colors on polygons.

A flexible overview of encoded units within a limited view of map space is required to clearly interact with and explore multiple visualization units with spatiotemporal features in a reasonable manner. The NSW COVID-19 statistics interface only supports inspecting the confirmed cases counted in each spot in the past month; also, it only shows the trend of data changes rather than allowing for numerical comparisons of various geo-spots within a constrained area or attribute comparisons of map overviews across different timelines.

- *R2: Record encoded visualization units in various analysis results* To support the user-centered exploration of historical and current results, implementing the storage function is needed to allow users to record the results of each interactive exploration and the relevant map parameters for later callback. Simultaneously, multiple storage steps can break the geographical barrier by allowing two units that are far apart to be checked and to track and compare temporal features underlying different timelines.
- *R3: Facilitate detailed comparisons of each encoded visualization unit in user-centered scenarios* One of the difficult parts of the comparison task is comparing detailed data that varies in both time and location. It should be capable of facilitating detailed comparisons of the same geo-location underlying different time phases, as well as data attribute value comparisons of multiple geo-locations underlying the same time phase. The timeline should include multiple categorized options.

## Visual design

To allocate the design requirements outlined in Sect. 3, we proposed a visual design, which employs visualization units for user in-process exploring and comparing spatiotemporal datasets. Any spatiotemporal datasets with spots in geographic coordinates and numerical values in time-vary attributes for each spot can be applied to this design easily. The visual design of each units is inspired by the Sunburst (Stasko and Zhang 2000) and Off the Radar (Albo et al. 2015) visualization of flower-like metaphors (Dong et al. 2020).

**Fig. 1:**
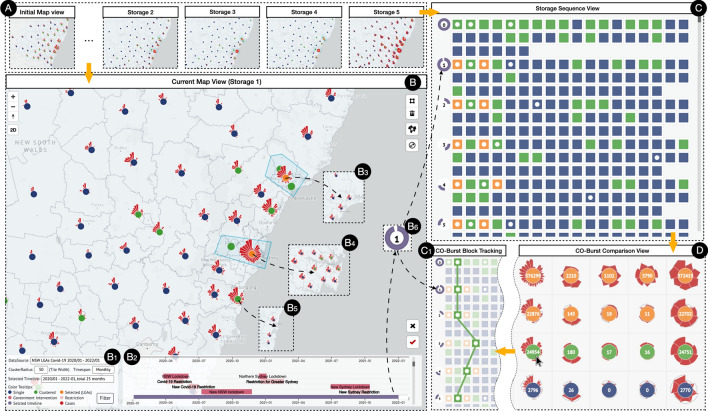
The interface of User-centered Visual Explorer (UcVE): (**A**) An inventory of user-stored different map views in sequence; (**B**) The current map view with the following components: map navigation buttons (left top), customize toolkit including select, cancel, expand and hide windows (right top), and store buttons, including delete and save functions (right bottom), (B1) Control Panel, (B2) Timeline with categorized events, (B3)-(B4) Multiple Custom-merged Spatiotemporal Visualization Units, according to place of interests, (B5) Auto-clustered Spatiotemporal Visualization Unit, (B6) Time span indicator: for each user-stored exploration result; (**C**) Storage sequence view: Overview of user-stored exploration history, and detailed visualization results in (C1) Block Tracking View; (**D**) Comparison View: supports comparing multiple user-selected visualization units and their data attribute values

**Fig. 2:**
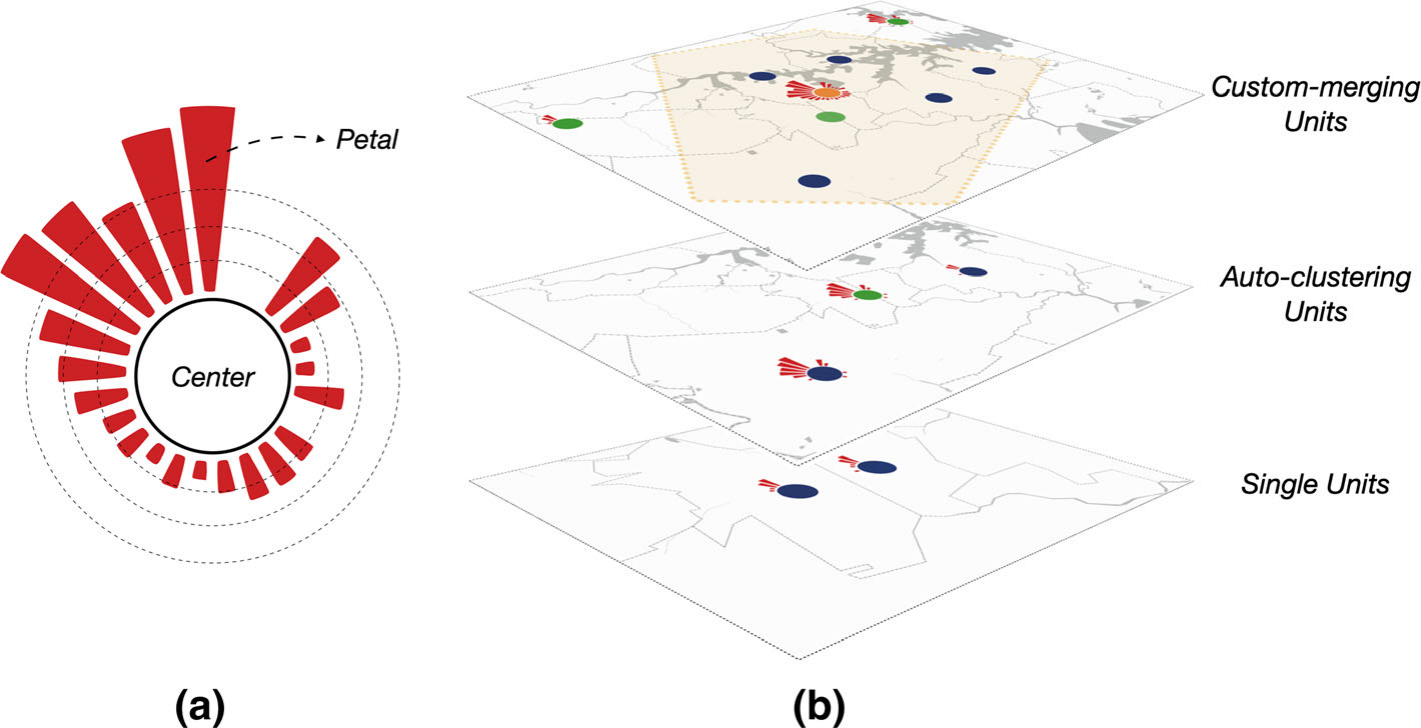
The design of visualization units. **a** A basic visualization unit with encoded petals and color-able center; **b** Three exploration scenarios of visualization units based on varying scales: single units, auto-clustering units and custom-merging units

In this section, we demonstrated the visual design of units, as well as user-centered progressive exploration strategies with UcVE Storage and UcVE Matrix interactions. (Fig. 1). For each distinct unit, we abstracted the spatiotemporal features into petals, and distinguished three statuses of visualization units by different colors in centers, as shown in Fig. 2a. The visualization units with a single unit (navy color), visualization unit with auto-clustered units which is automatically clustered according to different zooming scales (green color), and visualization unit with manually merged units (orange color) are defined based on current exploration scenarios, as shown in Fig. 2b.

### Visualization units design

We investigated redesigning each spot as a visualization unit with timeline attributes and mapping them to each geo-location to fully describe spatiotemporal features. The visualization unit should enable converting each unit into a single unit on the map, allowing each node to depict spatiotemporal features with a compressed adjustable time span.

#### Defining single visualization unit

The circular node is commonly used to pinpoint location information on a map. We locate the centroid of the polygon area of each target unit as geolocation of a single spot. To symmetrically attach temporal attributes, we redesigned the radial directions of the nodes for visualization. The x-axis of the polar coordinates is defined as the perimeter of a circular node, whereas the y-axis is defined as the vertical direction. Simultaneously, the perimeter of each node is sliced into a predetermined number of identical-sized spans to meet the visual discrimination channels of the user. Simultaneously, the attribute values of the corresponding time spans are allocated to each slice of the perimeter in a radially outward direction with different histogram heights (named petals). The formula for calculating petal *i*’s height in a single unit *s* is defined as follows:

1 $$\begin{aligned} \begin{aligned} P{(s_i,w)} = a \cdot \ln (F(s_i,w) + b), \,\,\,\,\,\,\,& \\ {F(s_i,w),a,\text {and }b \in N^*} \end{aligned} \end{aligned}$$

A growth petal height $P{(s_i,w)}$ of this visualization unit which refers to a single unit is proportional to the attribute value, in which ${F({s_i},w)}$ is the function to count the numerical value corresponding to this week *w*. Both *a* and *b* are positive integer parameters that can be adjusted to ensure that the petal height is *0* when the infection number is *0*.

#### Defining auto-clustered visualization unit

When there are too many rendered nodes, the query results on the map frequently cause overlapping issues, resulting in not only difficulty in recognition, but also vastly increasing the client’s rendering cost. We employed a progressive map node clustering method to solve this problem, which can automatically cluster nodes based on the provided clustering radius and regenerate a new cluster center to depict multiple nodes within the acceptable visible area, depending on the map’s current zooming scale. The function in *Equation (2)* is applied to calculate the petal *i* in week *w* of clustered visualization unit *c* which is clustered by other singles.

2 $$\begin{aligned} P{(c_i,w)}=a \cdot \ln \left( \sum \limits _{i=1}^{n}{F(s_i,w)} + b\right) \end{aligned}$$

The recalculated cluster centers should be biased toward the denser areas of the children units, and the clusters under different zooming scales based on map tiles should be weighted separately, so a step-weighted coordinate calculation should be applied.

#### Defining custom-merged visualization unit

Similarly, the heights of the petals in each clustered node should be reunited, but because simple superposition would result in excessive heights, we reframe the merged petal heights while merging the different statuses of nodes’ location sets. At this point, the merged petal height represents the sum of the infection cases in each time span of all the clustered nodes. After solving the node overlap problem by revising the clustering method, we realized that the automatic clustering method can sometimes result in geographical classification errors, which will hinder the user’s ability to explore target nodes, so we developed a visual analysis interaction based on drawing a polygon selection to merge customized aggregation.

As a result of the merge, the merged visualization unit *m* calculates the center and each petal’s height using a similar algorithm which sums each time span of infection cases in each merged visualization unit, to the units’ set and marks the centroid of the newly merged polygons as its location on the map. The petal *i*’s height of the merged unit is defined as follows:

3 $$\begin{aligned} P{(m_i,w)}=a \cdot \ln \left( \sum \limits _{i=1}^{n}{F(s_i,w)}+\sum \limits _{j=1}^{m}{F(c_j,w)} + b\right) \end{aligned}$$

where $P{(m_i,w)}$ represents the petal height in week *w* of the visualization unit merged by other clustered and single visualization units.

Any polygon *G* can be divided into *n* finite simple triangles $T_1,T_2,\ldots T_n$ where the centroid of these simple triangles is $(T_{i_x},T_{i_y})$ and the area is $A_i$. So the coordinates of the centroid of the new merged polygon are ($G_x, G_y$).

4 $$\begin{aligned} G_x= \frac{\sum {C_{i_x} \cdot A_i }}{\sum {A_i}}, G_y= \frac{\sum {C_{i_y} \cdot A_i }}{\sum {A_i}}, \end{aligned}$$

A brushable timeline linked with visualization units should be attached to help filter time phases more efficiently. Each visualization unit utilizes the adjustable segmented circular arc as the time span unit, with radially arranged petal heights displaying the attribute values in this time span.

However, because of the geographic distance between nodes and the radially grown petal directions, comparing the petal height of different visualization units will be more difficult. To demonstrate the differences among visualization units, we introduce comparison strategies in other visualization designs and interactions.

### Visual exploration strategies

The comparison strategies guide users on how to support exploring and comparing analysis tasks. The majority of geographic visualizations aim to output analyzed results while users interact with a map in diverse manners. However, the interaction results displayed by the map usually vary in different states. To effectively solve this problem, we implemented a user-centered visual explorer (UcVE) based on geo-map with viewing, saving, and tracking in-process visualization results.

#### UcVE storage: ranking

Considering that the user may vary the zooming scale or time phase of the view when exploring the map, we allow the visualization unit states of the current perspective to change.

The visual storage should support the saving of all visualization units and parameters set on the map during each step of exploration for easy reloading later.

Each visualization unit is compressed into a colored block and queued into a sequence with all units on the current map view. Considering different units occurs based on different map scale, we encode each block with a nested block to indicate belonging relationships. For example, block A is made up of blocks B, C, and D, and we have recorded A in sequence 1, B, C, and D in sequence 2. If we want to track B, the nested block in A will be highlighted with the same color as B. Both Figs. 4, 5,  6 and 7 show this situation.

Each storage step records each of the user interaction results in an ordered sequence, which is then ranked from left to right in descending order of each visualization unit’s total attribute value sum, and compresses each visualization unit into a nested-able block to depict the containing relations of units among different storage sequences.

We highlighted the associated visualization blocks that have the same spot names or contain spot names in different storage sequences. The time span indicator, which utilized the number of each storage sequence, is marked in front; the outer arc length of each number maps the time span of the storage and can be dragged to swap orders among other storage sequences, as shown in B${_6}$ in Fig. 1.

#### UcVE matrix: comparison

The UcVE storage function greatly increases users’ analytical scalability. To further compare the detailed value difference among visualization units, we introduce an $n\times m$ matrix space. Each grid in the matrix can visualize the detailed attributes of each visualization unit. The parameters *n* and *m* can be altered to accommodate users’ appropriate resolution. Each draggable grid is listed with comparable visualization units that aim to break through the limitations of objective factors (visualization units with different zooming scales or views, different time spans selected, etc.). However, in juxtaposition, it is difficult to precisely compare visual differences among items that are far apart, even if they can be adjusted in order. Therefore, we employed an explicit encoding on visualization unit that can extract and represent visual critical differences among juxtaposed visualization units in grids, rather than blinding users to minor changes in their comparison. The comparison strategy of each visualization unit in the grid is a benchmark peer-to-peer visual analysis method. Specifically, this comparison strategy involves selecting one visualization unit as the compared benchmark interactively, while other visualization units automatically vary based on the compared benchmark, and the encoded visual results of the changes show the difference in comparison.

This comparison strategy allows for highlighting the associated petals of the compared visualization units that correspond to the petals of the selected benchmark, as well as encoding the petal shape (raised or depressed) in other compared visualization units to show the compared differences. We listed two pre-options to encode the petal shape: Bezier curve and Polyline.

**Fig. 3:**
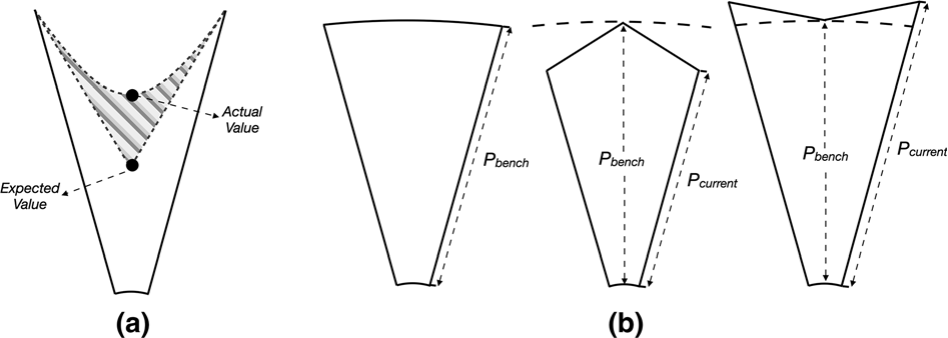
The comparison strategies in UcVE Matrix design. **a** It will cause value bias when using a Bezier curve to depict the petal’s outer shape; **b** It shows what is possible when using Polyline to plot visualization unit petals to compare the value differences

A Bezier curve, which is usually intended to approximate a real-world shape, is defined by a formula that uses a set of discrete control points to define a smooth, continuous curve. The definition of a Bezier curve indicates that the first and last control points are always the endpoints of the curve. However, the intermediate control points generally do not lie on the curve. This means that if the Bezier curve is used to describe the petal’s outer shape, the expected accuracy may not be depicted, as shown in Fig. 3. Therefore, we rule this option out.

The Polyline, in contrast to the Bezier curve, uses an accurate value to better describe raised or depressed differences. We defined that the radius of this petal at its highest or lowest point equals the radius of the compared benchmark petal $P_{bench}$, the current petal height $P_{current}$ describes the height of this petal edge, as shown in Fig. 3.

Furthermore, we added numbers and time span units to the colored center of the visualization unit to indicate the exact value of the entire visualization unit or one specific petal when hovering over it.

## COVID-19 as spatiotemporal dataset example

Spatiotemporal data relate to both space and time (Han et al. 2012). There is typically various information at various times on every spatial spot, and this information is typically accompanied by multi-dimensional attributes. When users encountered the tasks of comparisons among them, variation can bring analysis difficulties. As a result, it is crucial to separately represent the features of various dimensions, including time and space.

In the past two years, we have engaged in research on COVID-19 data in New South Wales (NSW), Australia. Supported by domain experts, we designed different visual tools to reveal useful patterns behind the basic statistics. During these collaborations, we noticed that the uncertainty of the COVID-19 epidemic means that traditional data analysis performs more poorly than effective visual models. In particular, the spatiotemporal features in hot spots, as inseparable parts of the epidemic analysis, played a significant role in analyzing the COVID-19 spread. The following subsections present COVID-19 as our data source, followed by variables of consideration corresponding to the design requirements.

### COVID-19 data sources

The NSW Government has made available open-source datasets for COVID-19 cases and tests within NSW (Data 2021). Our example dataset provides information on the number of daily infections based on COVID-19 case locations since the first infection was detected in Jan. 2020 to 2022. It excludes 189 cases of crew members who tested positive while onboard a ship docked in NSW at the time of diagnosis. The case aspects include confirmed, tested, recovered, and death by their notification date, location, age range, and likely source of infection, but the analyzing task becomes complicated because the available data variables are continuously adjusted and reduced due to privacy and other reasons. In addition, relevant news articles, alerts, and ministerial media releases issued by the NSW government on COVID were retrieved as events that can be combined with the notification date of the case dataset. As common division methods of usual residence, 128 Local Government Areas (LGAs) and 964 postal zones within NSW boundaries were included in the geographical datasets provided by the NSW Data Analytics Center (DAC), which were introduced with geo-polygon and geo-coordinates data.

### Variables consideration

As domain experts guided us to further meet the requirements, we discussed relevant key variables selected from multiple datasets.

Our COVID-19 case dataset contains information collected from January 1, 2020, to January 31, 2022, in NSW on cases by notification date and postcode, local health district, LGA, and likely source of infection. Due to the risk of personal information being leaked, only the notification date of confirmation and the location of each case’s usual residence can be accessed in this dataset.

During the data cleaning process, we discovered that issues arose when the same case corresponded to multiple postal zones; the 648 postal zones resulted in overcounts, making identification more difficult. The LGA, which is defined as an official spatial unit that may contain multiple postal zones, represents a reunited geographical neighborhood. Therefore, using LGAs can help to solve the situation where the same case is counted multiple times. Simultaneously, it can improve the map’s construction and readability. As a consequence, we chose 128 LGAs as the geo units for the aggregated area.

Considering that different government-released events caused different degrees of impact on the pandemic spread, we organized the retrieved media information of government-conducted events into two categories: intervention and restriction. Each time span unit was considered to cover all signature events in the past 2 years, which consisted of monthly cases summed up daily and listing 25 months of case data from each LGA.

In this study, we finalized 98298 rows of 25-month infection case data from Jan. 1, 2020, to Jan. 31, 2022, which we bridged with 2 categories of 12 significant events from 128 LGAs.

## Visual exploration workflow

**Fig. 4:**
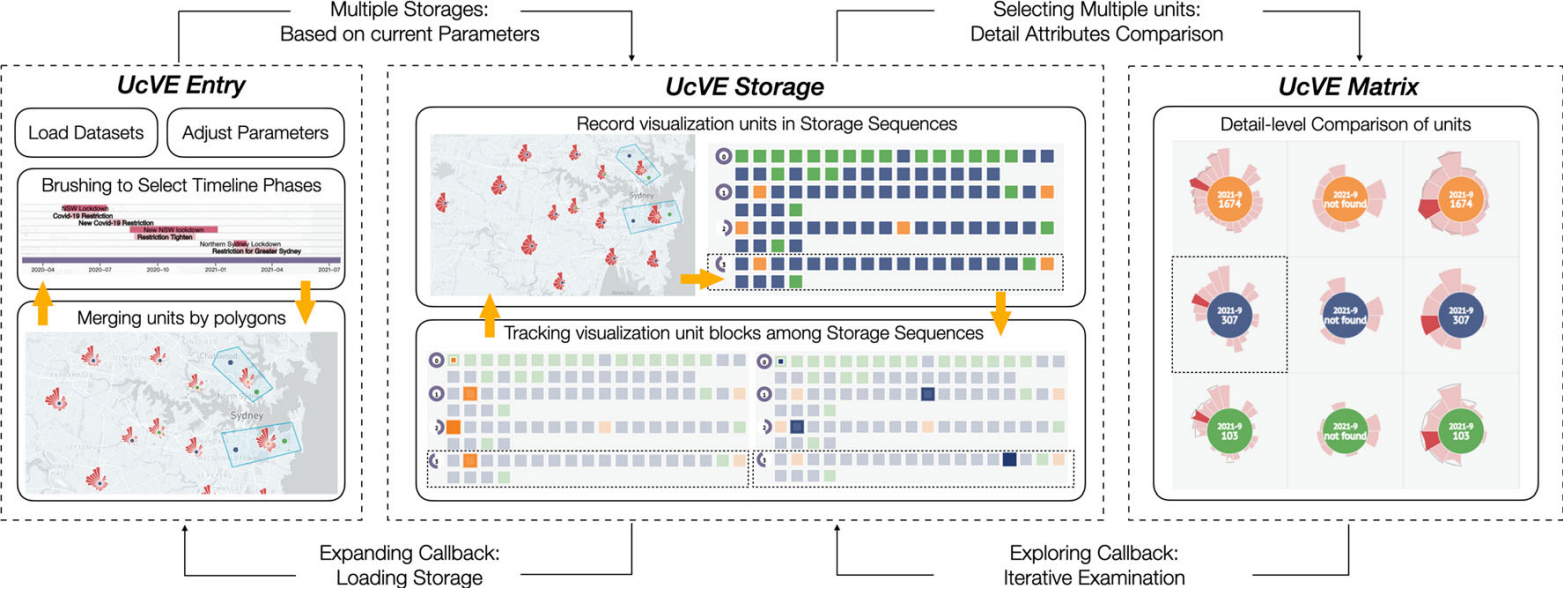
The entire workflow of UcVE embodies a user-centered progressive and comparative exploration, which consists of UcVE Entry, UcVE Storage, and UcVE Matrix

In this section, we propose a user-centered progressive workflow for the interactive visual exploration based on D3.js (Bostock et al. 2011) and Mapbox (Mapbox 2022), and further implement a visual analytics system applied to the COVID-19 datasets in NSW, Australia, to demonstrate UcVE from a workflow perspective, as shown in Fig. 4 .

### UcVE entry: parameters setting

After the visual analytics system loads the COVID-19 data, the user interacts by setting the parameters of the clustering radius and basic time span unit. The value of the clustering radius equals the width of a tile. The tile on the map is defined as a segmentable basic unit on the current zooming scale; the basic time span unit specifies the time span of the minimal unit represented by each petal in each visualization unit. Users can transfer multiple visualization units and all parameters for further exploration and comparison as entries to UcVE storage in sequence, including cluster radius, time span unit, current map view and zooming scale, and all unit data in the selected time span.

### UcVE storage: iterative exploration

The scalabilities are expanded by the diverse permutations and combinations of the transferred parameters to represent the various visual exploration results of multiple visualization units in which the user performed multiple storage steps. Users can iteratively zoom in or out to different scales to expand or recluster target units, as well as produce storage of multiples by adjusting the selected time spans. Simultaneously, the ranking strategy in UcVE storage supports tracking and highlighting each visualization unit into associated block that refers to the same or contains spots in different storage sequences. Moreover, each historical or current storage sequence can be reloaded on the map as a callback to reflect all the visualization units’ situations in this storage sequence.

### UcVE matrix: detail-level comparison

Users can drag and drop interesting visualization unit blocks into the UcVE matrix view for detailed comparison. Users can compare the similarity-difference patterns showcased by the encoded petals’ outer shape in this matrix. (1) Visualizing one unit within a selected time phase; (2) Tracking the varied trends of one specific unit in different time phases; (3) Listing the detailed attribute distribution of different units in different time phases; and (4) Comparing the detailed attributes of the same time span among multiple units are some of the possible patterns that can be explored or compared. Users can also progressively explore until they reach the conclusion by iteratively removing any visualization unit and adding new ones from multiple storage sequences.

## Case studies

In contrast to more sparsely populated areas, the coastal areas of NSW are more densely populated, making them more susceptible to cluster infections. For instance, over the last two years, the government has conducted periodic intervention and control events in all or parts of New South Wales, which may have suppressed the epidemic infection at various times.

In this section, we present three categorized cases: map-driven, storage-driven, and matrix-driven, which we progressively employ to explore and compare important connections and differences between spatiotemporal properties in the NSW COVID-19 dataset, as well as attempt to find the COVID behind the influence of LGAs on potential pandemic patterns. Relevant parameters must be pre-entered into the visual analysis system. In these case studies, users can customize the clustering radius, which corresponds to the tile width in the current map perspective, and the basic time span is set to monthly. The number of blocks which are used in each storage sequence depicted in each row is 18, and the size of the UcVE comparison matrix is 3 rows by 5 columns.

### Map-driven cases

In the initial map view, users can observe both automatically clustered visualization units and individual units, which are distributed on the map based on their geo-location. Users can adjust the zooming scales by zooming in and out and custom-merge their target visualization units by interactions to gather new visualization units of different statuses for map-driven case exploration. With the help of the brushable categorized timeline, users can adjust different time phases to filter visualization units on the map. In these map-driven cases, the clustering radius is set to 70 to better distinguish auto-clustered visualization units.

#### Visualization unit comparison of coastal LGAs with inland LGAs

**Fig. 5:**
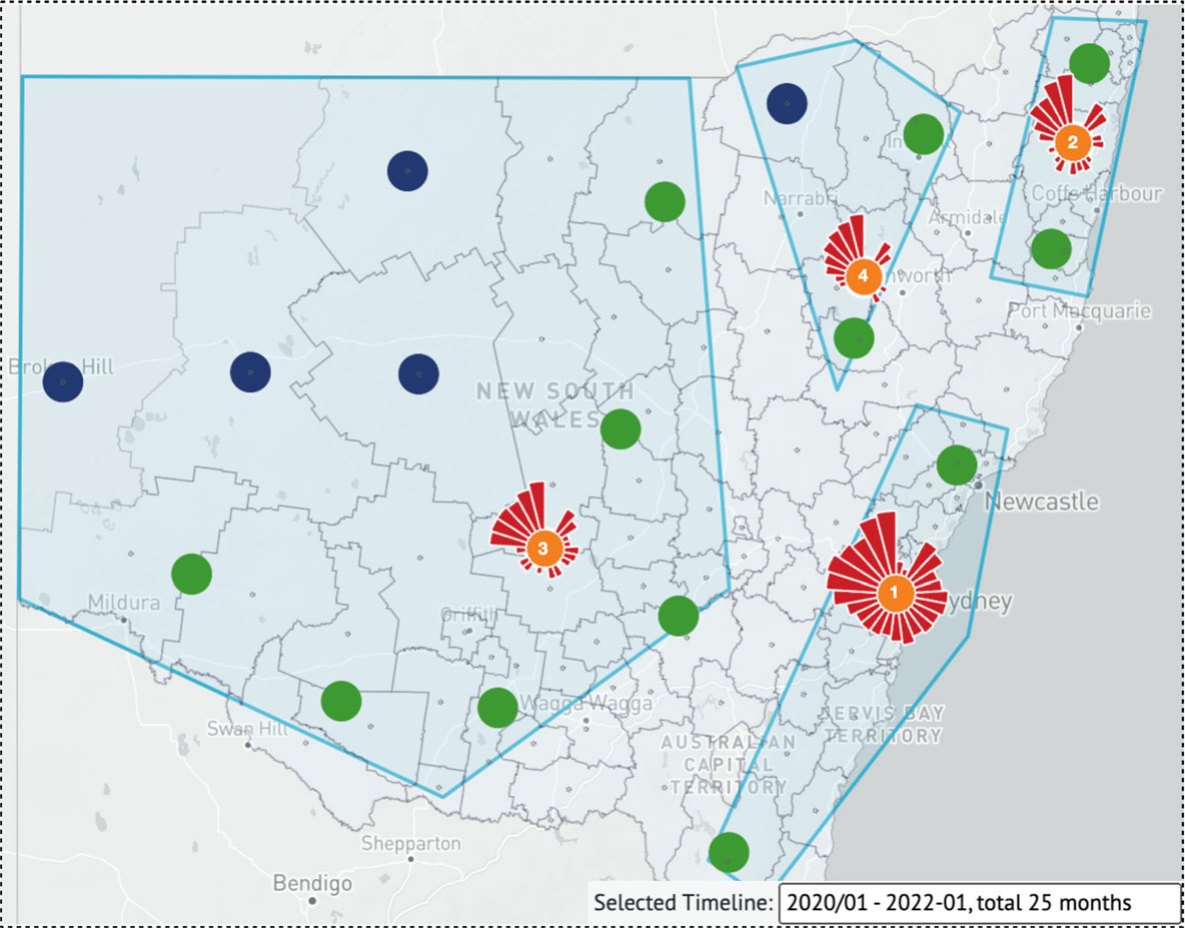
A map-based case with rankings in each visualization unit center. Using the interactive merge function, the 128 LGAs were classified into 4 visualization units representing the southeast coastal areas near Victoria (Rank 1); the northeast coastal areas near Queensland (Rank 2); the north-central areas (Rank 3); and the remote western areas (Rank 4) within NSW for exploratory analysis

We roughly divide NSW into two sections, coastal and inland. To better display visualization units on the map, we use the merged interactive tool to select four areas, two coastal and two inland as follows: the northeast coastal area near the border of Queensland; the southeast coastal areas near the border of Victoria, which has representative cities like Sydney, Newcastle, Wollongong, etc.; the north-central areas; and the remote western areas, as shown in Fig. 5.

When users look at the entire timeline over 25 months, they can observe that both the two parts of the coastal parts are ranked top 2 with the total number of COVID-19 infection cases in the whole period of 25 months, which is specified as the southeast coastal areas near Victoria win the first with merging 3 auto-clustered visualization units; then, the northeast coastal areas near Queensland reached the second with merging 2 auto-clustered visualization units, and followed by the remote western areas containing 6 auto-clustered visualization units and 4 single visualization units, and accompanied by the north-central areas with 2 auto-clustered visualization units and 1 single visualization unit.

The reason for such a wide spread of COVID-19 cases may be due to the geographical features of New South Wales. New South Wales is Australia’s most populous state, with a large number of densely populated cities along its coastal sides; rich forest resources in the north-central areas; and less populated remote western areas. We know that the epidemic’s spread is directly proportional to population density. This explains why COVID-19 outbreaks are more severe in densely populated coastal areas, despite the fact that the LGA area size is much smaller than in remote western areas.

#### Visualization units comparison of Northern and Southern Sydney LGAs

**Fig. 6:**
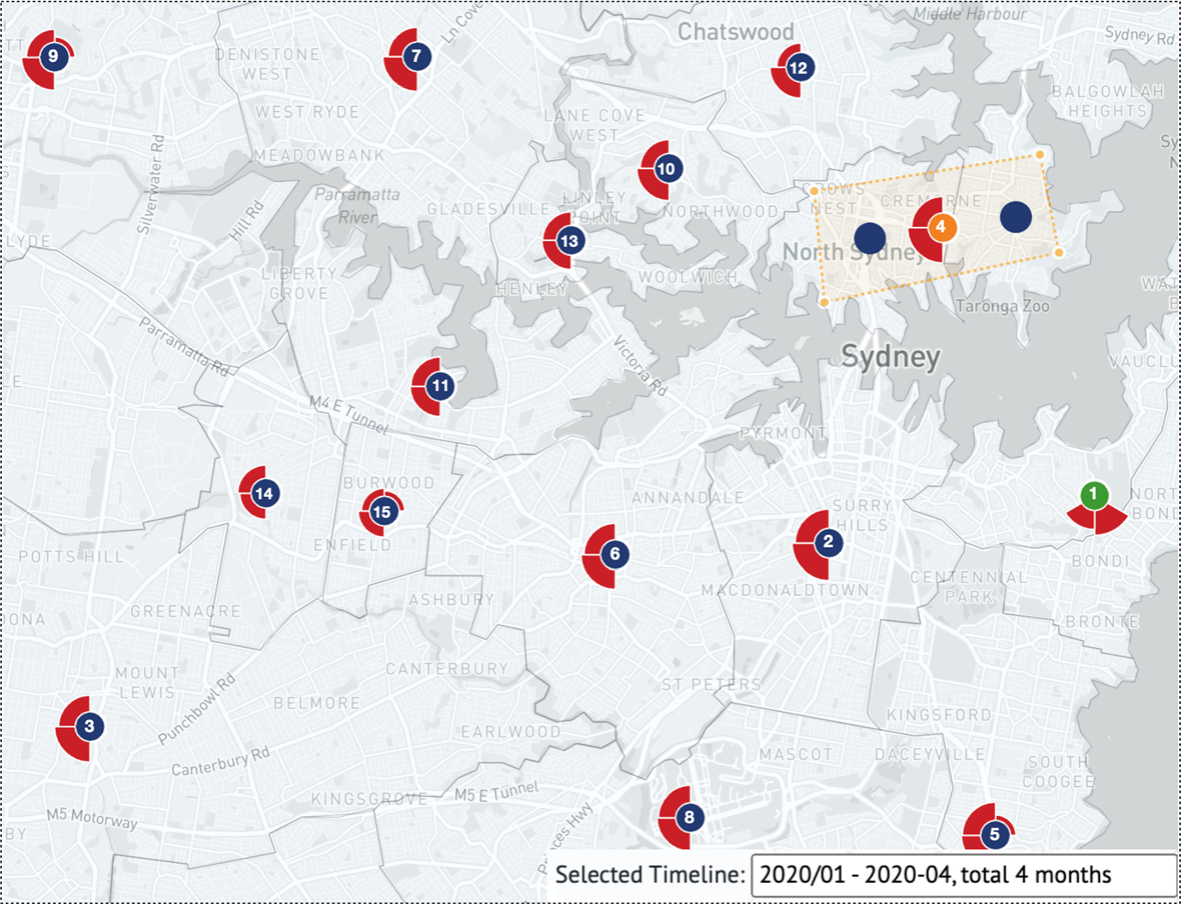
A map-based case with rankings in each visualization unit center. The first 4 months of the full timeline month (January 2020 to April 2020); merged Northern Sydney and Mosman from the perspective of the Sydney city map to explore and analyze the epidemic situation in northern and southern Sydney

Users can brush to select time phases by swiping through the entire timeline. We chose the first 4 months of the COVID-19 outbreak in this case to investigate the epidemic situation in Sydney and surrounding cities. We zoomed into the bottom-most visualization units displays around Sydney, then merged North Sydney with its adjacent neighbor, Mosman for further analysis on the map, as shown in Fig. 6. According to the petals of single visualization units representing Ku-ring-gai, Burwood, and Parramatta LGAs, we found that they had the first local cases in January; also, the ranking of the total number of confirmed cases found that visualization units representing Woollahra and Waverley had the highest total number of infection cases among the 16 visualization units around Sydney over the selected 4 months.

We note that the LGAs where the first local COVID cases occurred in January 2020 were Ku-ring-gai, Burwood, and Parramatta. These three LGAs are all major residential areas in the Greater Sydney District, which also proves the hypothesis that COVID-19 is prone to outbreaks in these places; and North Sydney and Mosman are the main business districts in Sydney. Compared with its southern neighboring LGA, Sydney (different from the Greater Sydney District), the north of Sydney is the main working area, while the south of Sydney is mainly for entertainment, with a large number of shopping malls and famous beaches in Bondi. As reported on various news outlets, after the Australian wildfires had been contained at the end of 2019, large crowds poured into beach destinations for a vacation, resulting in an extremely high population density, which became the source of the spread of the COVID-19 outbreak in early 2020, indirectly leading to the first lockdown in NSW.

### Storage-driven case

Multi-segment time phases can be divided by brushing the timeline in the 25 months and recorded into the storage view for tracking and the exploration of the number of COVID-19 cases represented in visualization units’ petals. In these storage-driven cases, the clustering radius keeps the set at 70.

#### Tracking the ranking of multiple visualization units

**Fig. 7:**
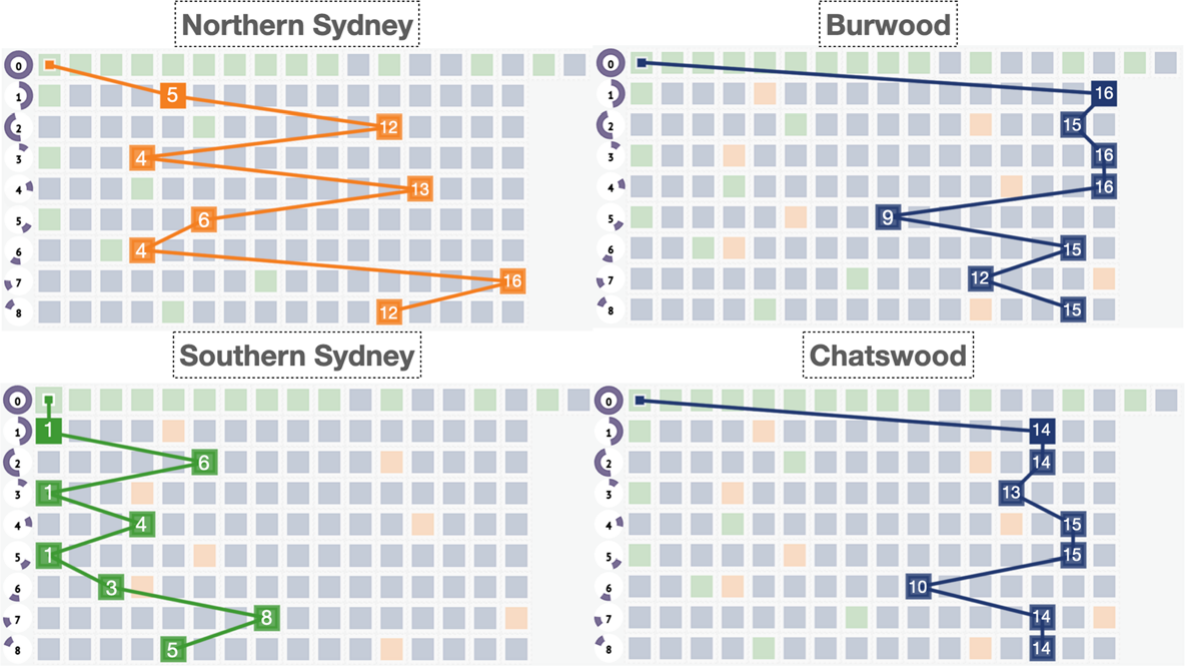
The storage-driven case. For visual analysis of their COVID-19 case rankings through two timeline classifications, four representative visualization units, Northern Sydney, Southern Sydney, Burwood, and Chatswood, were selected. Of these, the zeroth storage represents all visualization units stored in the overview state; the first and second storage steps respectively represent the epidemic data for the years 2020 and 2021 under the zooming scale of Sydney City; the third to eighth storage steps, respectively, represent the storage of visualization units in four-monthly time span units from January 2020 to December 2021

We drew a polygon to select the entire Greater Sydney Districts from the overview and classified the whole timeline into two options. One option is to divide the entire timeline into two years, 2020 and 2021; the other is to divide the entire timeline on a 4-monthly scale, shown in the time span indicator. The ranking track of each using the storage function to record their visualization units in batches is shown in Fig. 7.

We selected 4 representative visualization blocks, namely Northern Sydney, Southern Sydney, Burwood, and Chatswood, from those recorded for comparative analysis. Our observations reveal that the four blocks are all from the first visualization block in the zeroth storage, which is the Sydney LGA’s visualization unit block with the largest number of COVID-19 cases at the time of the initialized overview. Sydney’s epidemic performance is worse than Burwood and Chatswood from an overall perspective, whether in years or in a 4-month phase. Analyzing the growth trend, we found that Northern Sydney, Southern Sydney, and Burwood all showed signs of a rebound in the number of COVID cases from August 2020 to December 2020, while Chatswood experienced a rebound in cases from December 2020 to April 2021. Based on the combined NSW government interventions and restrictions, it was found that during the two lockdown periods (March 2020 to June 2020 and July 2020 to November 2020), there was a short period of unblocking, which led to the second wave of the COVID epidemic, and after the second lockdown in November, the third spread of Omicron.

Among their own visualization blocks, the total case rankings in the Northern Sydney region fluctuated greatly, while the Southern Sydney region was always in the top 8; on the contrary, Burwood and Chatswood never entered the top 8. We also combined official information from NSW census data (Statistics 2016) to find that COVID-19 spread quickly in crowded residential areas, such as South Sydney, Burwood, and Chatswood, which are popular tourist destinations, and led to a secondary transmission of the virus when the lockdown period ended whereas, in North Sydney, which acts as a business district, the number of infections responded quickly to government interventions because people worked from home during the lockdown period.

### Matrix-driven cases

The visualization units in the matrix allow for comparison at a fine granular level. The user is not only able to select any visualization unit from the storage view, but they also enable examining the differences in each petal’s attribute values by observing the various petals’ outer encoded shapes.

#### A comparison of multiple visualization units before and after government interventions

**Fig. 8:**
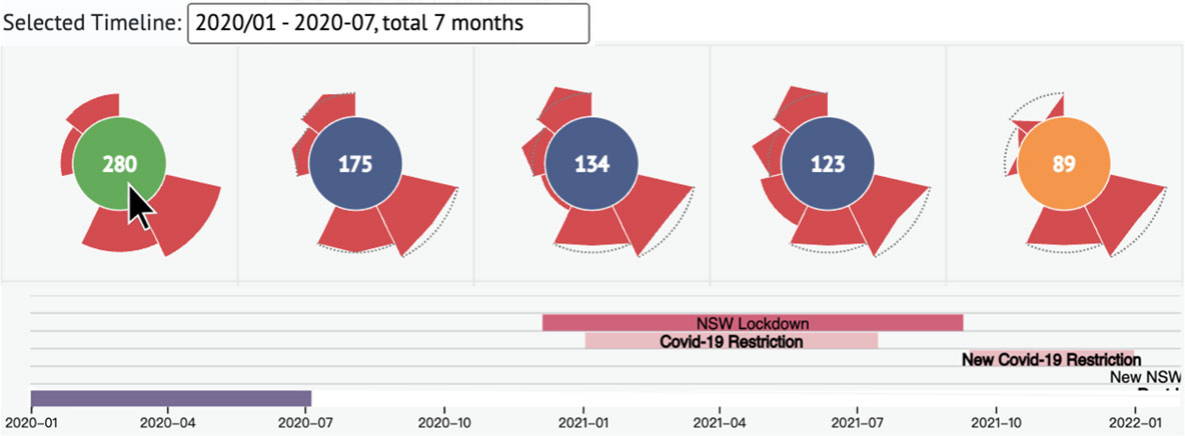
The matrix-driven case of exploring multiple visualization units before and after government interventions. Picked in order from the top 1 to 5 of the total infection cases in the zooming scale of the Greater Sydney Districts with the time phase from January to July 2020: auto-clustered visualization unit of Woollahra and Waverley, followed by 3 single visualization units, LGA Sydney, Canterbury-Bankstown, and Cumberland, and a custom-merged visualization unit of Northern Sydney and Mosman

In this case, the clustering radius is set to 70. The intended time period we brushed for this case starts from January 2020 to July 2020; then, we recorded the interaction results and picked the top 5 visualization units for a detailed comparison in this matrix after custom-merged the visualization units of Northern Sydney and Mosman. In terms of overall COVID-19 cases, the auto-clustered visualization unit (Woollahra and Waverley) had the highest number of infections, followed by three single visualization units (LGA Sydney, Canterbury-Bankstown, and Cumberland). The combined visualization unit of Northern Sydney and Mosman is fifth, as shown in Fig.  8.

By comparing the petal shapes in the coming months, we discovered that the auto-clustered visualization unit of Woollahra and Waverley has much lower cases than the other three single visualization units (in decreasing order, these being Cumberland, Canterbury-Bankstown, and Sydney) from May to July 2020. Moreover, Woollahra and Waverley had 0 diagnosed cases in May 2020, and only in June and July did these areas have more than the combined nodes of Northern Sydney and Mosman (Northern Sydney and Mosman had 0 infection cases in May and June 2020).

We note that the time phase we selected from January 2020 to July 2020 includes the vacuum period from January to March 2020, the first lockdown from the end of March to June 2020, the related restriction in June 2020, and the starting point of the second lockdown beginning in July. This visual finding verifies the earlier case analysis that a huge number of individuals flocking to coastal areas acted as a catalyst for the epidemic’s early development. COVID-19 spread when the government began intervening with lockdown events in March, causing new cases in the LGAs in residential communities. Because Northern Sydney and Mosman are both in the CBD, there were no new cases in the lockdown from May to July.

#### Exploration of multiple visualization units during government interventions

In this case, the clustering radius is set to 50. Due to the vast majority of government intervention events targeting the Greater Sydney Districts, we zoomed into the view of the Greater Sydney Districts on the map. We found that the Waverley and Woollahra areas that were severely affected by the COVID-19 outbreaks in the early phases were not automatically clustered together, so at that time, we used the merge function to merge them as a new visualization unit. In the current storage sequence, we found that the custom-merged visualization unit (Waverley and Woollahra) did not appear in the top five, and we clicked to select the top five visualization units for a detailed comparison. We hovered the mouse over each petal of the first Canterbury-Bankstown, and the petals of the other visualization units (Cumberland, Sydney, Bayside, Randwick, and Parramatta LGAs) were highlighted correspondingly. From June 2021 to January 2022, the comparison results represent the difference in the number of cases each month for the five selected visualization units.

**Fig. 9:**
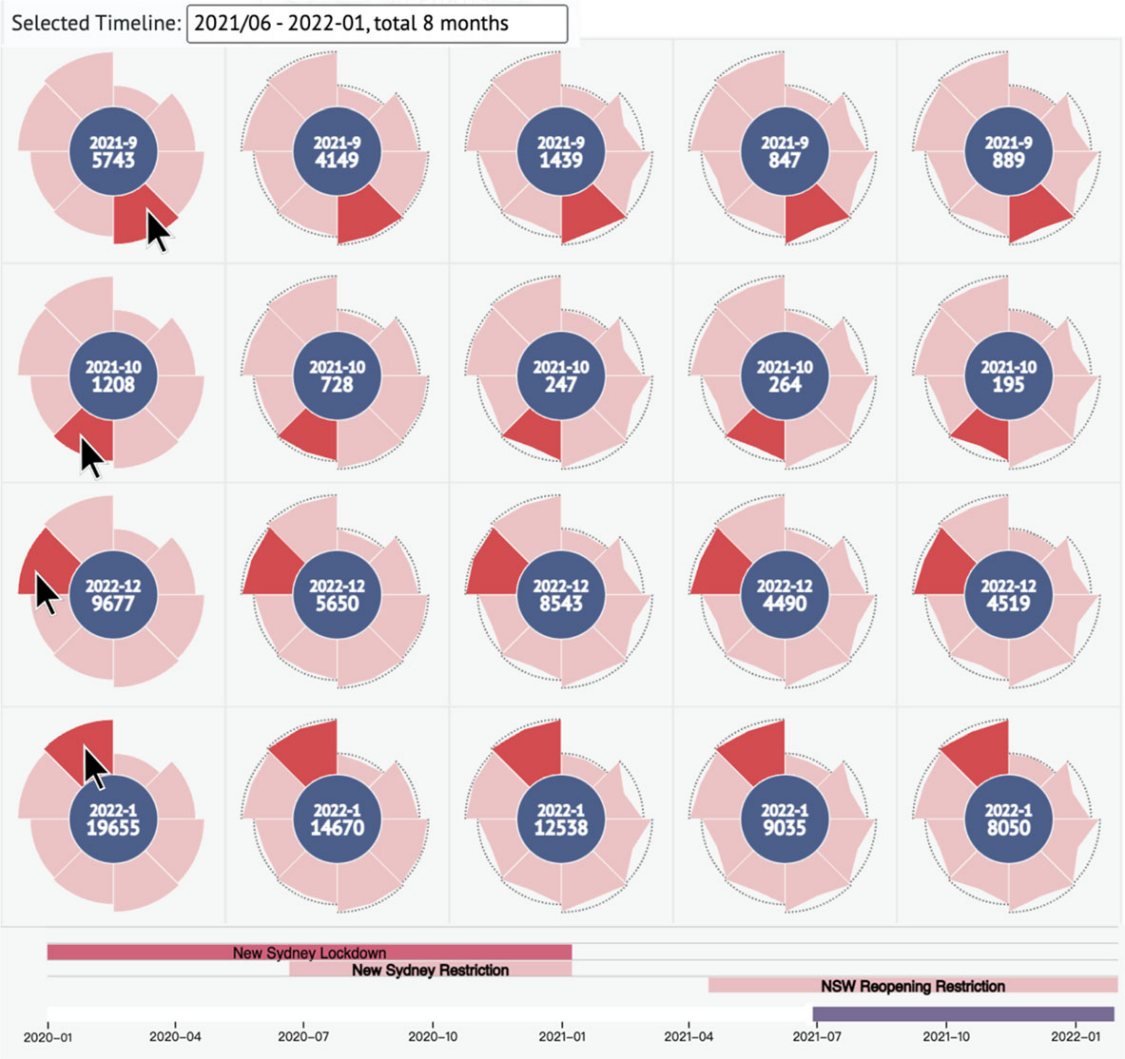
The matrix-driven case of multiple visualization units during government interventions. Picked in order from the top 1 to 5 of the total infection cases in the zooming scale of the Greater Sydney Districts with the time phase from June 2021 to January 2022. The selected visualization units represented LGAs from left to right are Canterbury-Bankstown, Cumberland, Sydney, Bayside, Randwick, and Parramatta

The results in Fig. 9 show that Canterbury-Bankstown had the highest number of COVID cases for each of these 8 months. Although in some months, the ranking of other visualization unit cases is not followed by the total ranking order from the total number of infections, for example, Sydney (with a total ranking of third) has 8543 confirmed cases in December, which is much larger than Cumberland’s 5650 (total ranking second), but overall, the ranking order of this selected phase is to a certain extent the same.

In line with its intervention measures, the NSW government announced the third closure of the Greater Sydney Districts in June 2021 to deal with the mutated COVID-19 virus, Omicron. During the lockdown phase, it can be seen that in the first half of the lockdown, that is, from June to September, the number of confirmed cases of the epidemic was still increasing gradually, and there was no downward trend until October, so the government ended the lockdown at the end of October. The epidemic was relatively stable in November after the lockdown ended, but during the Christmas holiday season in December, it increased and reached a peak in January 2022, with three single units in a single month, which is more than 12,000 cases.

## Discussion with domain experts

We deployed the visual analytic system based on UcVE online and discussed our case study findings with two anonymous domain experts from the Australian government in different departments.

After learning about our methodology and exploration strategies, both experts expressed their enthusiasm for using UcVE to analyze the spread of COVID-19 in NSW. We asked the experts to explore on their own using our system, answering their questions and recording their preferences and feedback. By redoing our case studies, they first affirmed our findings in the different cases before delving into various explorations based on their individual interests in the COVID-19 dataset. They mentioned that the UcVE-based visual analysis system was responsive and that the storage function was flexible enough to give them enough analytical confidence. They all appreciated our visual outputs in universal state that makes possible to connecting spatial-temporal to general analysis with rich adjustable parameters and grants possibilities on some very aggregate predictor questions. The expert from the NSW Health Department commented that the system not only displays the full range of COVID-19 with its geographic and timeline features, but it also presents the data in a clear and interactive visualization output. The other expert from the DAC, NSW, expressed the view that the comparison function of UcVE was initially difficult to understand. However, after learning, it was possible to actively compare the distribution of any one or more LGAs within NSW during the past two years. Three experts appreciated UcVE’s visual design and anticipated the future benefits of this technology, which allows for precise time comparisons over a single day or week.

## Limitations and future work

Our research is generally limited by two aspects. One being is that the spatiotemporal features may come from various data sources among different analytical tasks. For example, the COVID-19 datasets in NSW were applied to UcVE, which includes diverse data sources from official releases, social media, and unstructured textual data sets obtained by the researchers of this paper, which constrained our work. Manually integrating these datasets may produce inaccurate results. Relevant COVID-19 case data were collected by the NSW government based on case locations, yet some records failed to specify the locations where the infections were acquired. Moreover, although the COVID-19 case data released by the NSW government contain open-sourced postal areas and LGAs in the geographic information, we found that there are a lot of errors and null values in the postal area data, which prevents us from using a more detailed geo-scale to explore their locations.

Secondly, our work is also constrained by parameter settings. Our UcVE-based visual analytics system delivers strong scalability for user-centered analysis. However, it requires prior learning of how to use the system and how to set relevant parameters, which may incur additional learning costs. For instance, setting a proper clustering radius may make exploration easier; the size of the parameters of the visual cues in the storage and comparison matrix must be set by users based on their screen resolution; the parameters for calculating the petal height in UcVE need to be set based on data magnitude for a better display.

In light of these limitations and the feedback from the domain experts, we updated our system by expanding the diversity of related COVID-19 datasets and updating the supporting functions. We will take into consideration the NSW government’s follow-up updates and obtain more precise and thorough location data. For example, we can drill down from LGAs to postal areas and even construct a visual analytic system based on travel paths if permitted. Simultaneously, we will consider incorporating census data for each sampling location, such as local population and economic indexes, to better understand the relationship between these factors and the epidemic’s spread. We will also improve the system’s storage function to enable the collaborative exploration of multi-users online as well as an analysis of multiple users’ interaction behaviors. With these improvements, we also plan to conduct a systematic evaluation with more government staff. As suggested, we will offer an automated analysis function of real-time data, which would help relevant policymakers apply proper decisions.

## Conclusion

In this paper, we present UcVE, a user-centered visual explorer for in-process exploring and comparing spatiotemporal features by visualization units. The visualization unit uses a geo-based layout to display several visualization units by abstracting the attribute values of time-varying properties into encoded petals that compress at each unit’s geolocation. We adjusted the aggregation purposes to define three distinct visualization unit statuses: single, auto-clustered, and custom-merged, keeping map space efficient and enhancing user exploration capabilities while avoiding overlapping issues. The visualization unit allows users to visualize, save, and track in-process exploration results to reduce user cognitive load. We explain it as two exploration strategies: one preserves users’ visualization unit interaction output as steps in the current map perspective for historical and current exploration results callback, and the other allows users to compare the attribute values of temporal properties by the encoded petal outer shape of multiple visualization units at a detailed level.

We further implement a visual analytic system that offers UcVE for visual exploration and comparison of COVID-19-related datasets. We abstracted each LGA with a coordinated location and its monthly COVID-19 infection cases to encode on the map and coupled the map with a brushable timeline chart to filter different time phases. Simultaneously, two other charts in the system responded to two exploration strategies for tracking visualization unit blocks and comparing encoded visualization units in the matrix. We used three separate case studies in the COVID-19 dataset to demonstrate the efficiency of visualization units, each driven by a different function, and explored the causes of the visual results. We also undertook a review in cooperation with domain experts and prioritized our future work based on their feedback.
